# Neuropilins in the Context of Tumor Vasculature

**DOI:** 10.3390/ijms20030639

**Published:** 2019-02-01

**Authors:** Stephan Niland, Johannes A. Eble

**Affiliations:** Institute of Physiological Chemistry and Pathobiochemistry, University of Münster, 48149 Münster, Germany; johannes.eble@uni-muenster.de

**Keywords:** endothelial cell, neuropilin interaction partners, neuropilin ligands, neuropilin signaling, semaphorin, tumor angiogenesis, tumor microenvironment, tumor vasculature, tumor-penetrating peptides, vascular endothelial growth factor

## Abstract

Neuropilin-1 and Neuropilin-2 form a small family of plasma membrane spanning receptors originally identified by the binding of semaphorin and vascular endothelial growth factor. Having no cytosolic protein kinase domain, they function predominantly as co-receptors of other receptors for various ligands. As such, they critically modulate the signaling of various receptor tyrosine kinases, integrins, and other molecules involved in the regulation of physiological and pathological angiogenic processes. This review highlights the diverse neuropilin ligands and interacting partners on endothelial cells, which are relevant in the context of the tumor vasculature and the tumor microenvironment. In addition to tumor cells, the latter contains cancer-associated fibroblasts, immune cells, and endothelial cells. Based on the prevalent neuropilin-mediated interactions, the suitability of various neuropilin-targeted substances for influencing tumor angiogenesis as a possible building block of a tumor therapy is discussed.

## 1. Introduction

Cancer, one of the most common causes of death, appears in many ways, and is individually different in almost every patient. Although the causes of cancer are complex and elusive, it actually always arises in only three ways, i.e., by accumulation of genetic defects, by carcinogenic environmental factors, or by innate hereditary defects. Essentially, three key processes can be affected: (i) cell fate, (ii) cell survival, and (iii) genome maintenance. In their regulation, twelve signaling pathways are involved using approximately 140 genes [1]. Of particular importance for cell survival and cell fate is neuropilin (NRP), which is involved as a coreceptor of important receptor tyrosine kinases (RTKs) in STAT, RAS, MAPK, PI3K, Notch, TGF-β, and hedgehog pathways, as well as Wnt/β-catenin signaling. These signaling pathways are of paramount importance for vascular biology, as they are critically involved in angiogenic processes. Tumors often show altered NRP expression levels compared to normal tissues [2]. Viability and growth of various tumor cells is critically dependent on their NRP expression, as evidenced by its abundant expression in various advanced-stage tumors [3,4]. Therefore, interest in NRPs as new therapeutic targets is great. While the roles of NRPs in the nervous system and in the immune system have recently been reviewed elsewhere [5,6], this review focuses on the role of NRPs in the context of the tumor vasculature. It compiles old and new NRP interaction partners and discusses possibilities to intervene in NRP signaling within the tumor vasculature.

NRPs are involved in a wide variety of signaling pathways and have pleiotropic effects on axon guidance and remyelination, immune response, angiogenesis, cell survival, migration and invasion [7,8,9,10]. First, NRP-1 was characterized as a receptor for the class 3 semaphorin SEMA3A [11,12]. Soon afterwards, it turned out that NRP on ECs and tumor cells also binds Vascular endothelial growth factor (VEGF) [13], and that NRP, whose overexpression leads to leaky and hemorrhagic hypervascularization, is of paramount importance for proper development of blood vessels [14,15].

## 2. Molecular Structure of NRPs

**Figure 1:**
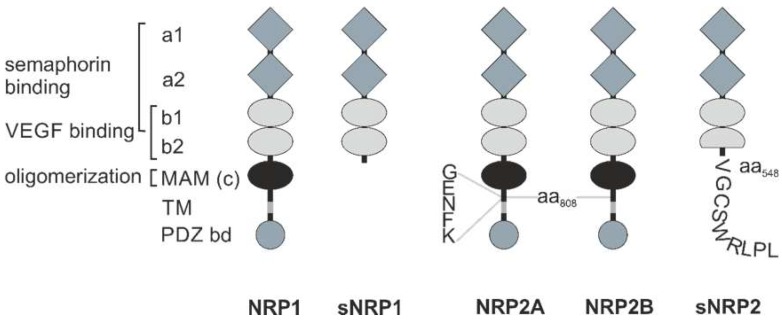
Schematic structure of the neuropilins. The extracellular portion of NRP1 has two Cubilin homology (CUB) domains, termed a1 and a2 (grey diamonds), two factor V/VIII homology domains, termed b1 and b2 (light grey ellipses), a linker region, and a meprin/A5-protein/receptor protein-tyrosine phosphatase mu, or for short, MAM or c domain (black ellipse). Via a single transmembrane (TM) domain (grey) it is linked to a cytoplasmic domain containing a C-terminal PSD-95/Dlg/ZO-1 (PDZ) binding domain motif (dark grey circle) with the characteristic amino acid sequence SEA. For semaphorin binding, the a1/a2 tandem domain together with the b1 domain is required. Binding of VEGF is mediated by the b1/b2 tandem domain. The MAM domain is necessary for oligomerization. Soluble NRP1 (sNRP1), of which there are four isoforms, is truncated C-terminally of the b2 domain. Despite different amino acid sequence, NRP2 has the same domain structure as NRP1. However, NRP2 differs from NRP1 by an insertion of five amino acids between its MAM and transmembrane (TM) domain. Soluble NRP2 (sNRP2) has a truncated b2 domain and nine additional amino acids at its C-terminus.

NRPs are evolutionarily conserved cell surface proteins that are expressed by all vertebrates and are widely distributed in adult tissues [13,16]. They form a small family, essentially consisting of two members: NRP1 is a 120 kDa and NRP2 a 112 kDa transmembrane glycoprotein [13,17,18,19,20]. The NRP1 and NRP2 genes are encoded at two different loci on chromosomes 10p12 and 2q34, respectively [21]. Due to RNA splicing, there are different membrane-bound and soluble splice variants of both NRPs; especially NRP2 comes in different variants, NRP2A and NRP2B (Figure 1) [22,23,24,25,26]. This diversity is further enhanced by optional insertion of 5, 17, or 22 amino acids C-terminal to amino acid 808 in the membrane-proximal part of the NRP ectodomain. The extracellular portion of NRP1 and NRP2 consists of two Cubilin homology (CUB) domains (a1/a2), two FV/VIII domains (b1/b2), and a meprin/A5-protein/receptor protein-tyrosine phosphatase mu, or for short, MAM (c) domain [16]. The CUB domains have significant homology with complement factor C1s/C1r, Bone Morphogenetic Protein 1 (BMP1), and Tolloid proteins, while the FV/VIII domains are homologous with the coagulation factor FV/VIII, a receptor-type tyrosine kinase DDR, and discoidin-1. The third (c) domain is often referred to as MAM domain, corresponding to the abbreviation of meprin, A5 (former name of NRP), and receptor-type protein-tyrosine phosphatase mu and kappa (PTPμ, κ) [27,28]. There are soluble NRP variants, sNRP1 and sNRP2, consisting only of these tandem a1/a2 and tandem b1/b2 domains, while lacking the MAM domain, as well as the transmembrane and cytoplasmic domains. The transmembrane domain spans the plasma membrane once and links the ectodomain with a comparatively short cytoplasmic region, which is 44 amino acids in length in the case of NRP1, and 42 and 46 amino acids in length for NRP2A and NRP2B, respectively. None of the membrane-anchored NRP variants has a cytosolic tyrosine kinase domain [16,22]. Instead, the variants, NRP1 and NRP2A, possess a PSD-95/Dlg/ZO-1 (PDZ) binding motif at their intracellular carboxyl termini [16].

Both NRP proteins can be glycosylated to varying degrees in different cell types [29,30,31]. Moreover, NRP1 and NRP2 show distinct glycosylation patterns. NRP1 is N-glycosylated and/or bears a glycosaminoglycan side chain at Ser612 [31]. This side chain is predominantly a chondroitin sulfate in vascular smooth muscle cells, whereas NRP1 on human umbilical vein endothelial cells (ECs) is equally glycosylated with chondroitin sulfate and heparan sulfate chains [29]. In contrast, NRP2 is not a proteoglycan, but rather belongs to a small group of proteins that can be polysialylated. Such a posttranslational variation of polysialylation is observed on dendritic cells, and it determines their trafficking to secondary lymph organs and their interaction with T-cells [6,32]. However, NRP1 can also be polysialylated, but only about half as strong as NRP2 [33]. The structural diversity of NRPs is further enhanced by proteolytic processing by ‘a disintegrin and metalloproteinases’ ADAM9 and ADAM10 in ECs. This results in membrane-anchored NRP isoforms that lack the extracellular a1/a2 and b1/b2 tandem domains, or even the MAM domain [34].

Two NRP molecules non-covalently associate into a homodimer, presumably via their MAM domain and via motifs within their α-helical transmembrane domain [35,36]. In particular, the α-helical transmembrane domains align and thus substantially contribute to homodimerization [36,37]. In addition to homodimers, NRP1 and NRP2 can also form heterodimers [38]. It is still unclear, whether dimerization occurs immediately after translation within the ER or during vesicular transport to the cell surface.

## 3. Tissue Distribution of NRP1 and NRP2

NRP1 and NRP2 are differently distributed in tissues. First identified on neuronal cells of the central nervous system, especially in the developing embryo, NRP1 was also found to be expressed in blood vessels of different tissues, especially in arterial vessels. In the earliest stages of development, NRP1 and NRP2 are differentially expressed in arteries and veins, respectively [39]. Later in ontogeny, NRP1 is expressed on arterial ECs and in the tumor vasculature, but not on venous or lymphatic ECs [40,41,42,43]. In adult tissues, it is highly expressed in heart and placenta, moderately expressed in lung, liver, skeletal muscle, kidney and pancreas, as well as in bone marrow stromal cells, osteoblasts, and keratinocytes, and lowly expressed in the adult brain ([13,44,45,46], https://www.uniprot.org/uniprot/O14786, accessed on: 23 January 2019).

NRP2, on the other hand, is expressed in neural crest-derived cells, such as hepatocytes and epithelial cells of proximal and distal renal tubules [47]. In the vasculature, it is restricted to ECs of veins and lymphatic vessels [41,42,43]. In particular, it is expressed by a subset of ECs during tumor lymphangiogenesis [48] as well as in tumor cells, where its function is still unclear [49]. Unlike NRP1, which is more likely to be expressed by carcinomas, NRP2 is rather expressed in neuronal tumors and in melanomas [50].

NRP1-knockout results in embryonic lethality due to severe vascular defects with missing capillary networks and unorganized blood vessels [15,51]. NRP2-deficient mice, on the other hand, are viable with a functional vasculature, but a severely reduced system of lymphatic vessels [51,52]. In addition, in osteoblasts and osteoclasts, NRPs are involved in regulation of bone homeostasis [53,54,55]. NRP2-knockout mice suffer from low bone density because of a decreased number of osteoblasts and an enhanced number of osteoclasts [53]. Both NRPs also show a distinctly different expression pattern on different immune cells [6,56,57]. While regulatory T cells (T_reg_) and basophilic granulocytes express NRP1, dendritic cells, macrophages and microglial cells express both NRP1 and NRP2, [58,59].

Pathologically, NRPs are expressed by tumor cells of various cancers, such as brain, breast, lung, colon, ovarian, and prostate cancers, and they contribute to tumor progression presumably due to their regulatory role in angiogenesis and in immune suppression [13,35,60,61,62,63,64].

In the last few months alone, expression of NRP1 has been reported in a variety of cancers, including oral squamous cell carcinoma [65], gastric cancer [66], pancreatic duct adenocarcinoma (PDAC) [67,68], hepatocellular carcinoma [69], cholangiocarcinoma [70,71], colorectal carcinoma [72], prostate cancer [73], and breast cancer [74]. Furthermore, among the sarcomas, leiomyosarcoma cells have been recently reported to express NRP1 [75]. Also, in melanoma [76,77] and in neuronal cancers, such as glioblastomas [30,78,79], NRPs are expressed and play a functional role in tumor progression. NRP2 is highly expressed in lymphatic ECs and in the stroma of many tumors, such as glioblastoma [78], oral and esophageal squamous carcinomas [80,81], salivary adenoid cystic carcinoma [82], thyroid carcinoma [83], breast cancer [84], gastric cancer [61], hepatocellular carcinoma [85], pancreatic cancer [86], colorectal cancer [87], bladder cancer [88], renal cell carcinoma [89], prostate cancer [90], osteosarcoma [91], lung cancer [92], and melanoma [93], where it promotes lymphatic metastasis by increased tumor lymphangiogenesis, and thus correlates with tumor stage, progression, and a poor prognosis [61]. Poor patient survival is also due to the fact that NRP2B expression correlates with immune cell checkpoint receptor ligand PD-L1 abundance, epithelial to mesenchymal transition (EMT), and acquired resistance to epidermal growth factor receptor (EGFR) inhibitors [94].

## 4. Potential NRP Interaction Partners: Extracellular Soluble Ligands and Trimeric Complexes with Signal Receptors

**Figure 2:**
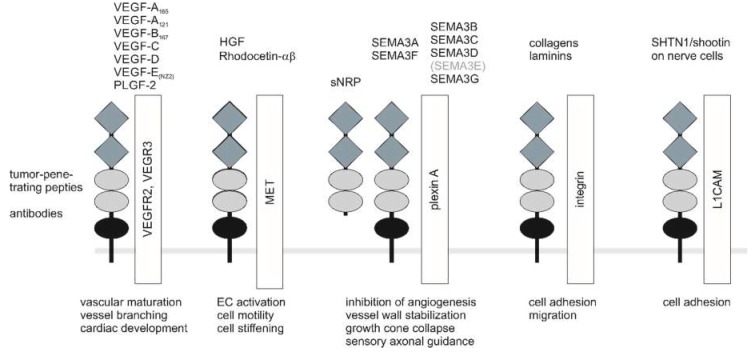
NRP coreceptors, ligands, and NRP-targeting compounds. For clarity, only monomers of receptors and coreceptors are shown. For signaling, dimerized ligands bind to a NRP dimer that interacts with a pair of receptor type kinases or plexin receptors. Soluble sNRP isoforms can interfere with the signaling of NRP-containing holoreceptors. Physiological NRP ligands involved in angiogenesis and tumor angiogenesis, vascular branching and maturation, as well as cardiovascular development, are VEGF-A_165_, VEGF-B_167_, VEGF-C, VEGF-D, VEGF-E, and PLGF-2. Among the semaphorins important for the nervous system, the secreted forms SEMA3A and SEMA3F as well as the membrane proteins, SEMA3C and SEMA3D, have important functions in the tumor vasculature. SEMA3E is the only semaphorin that directly binds to a plexinD1 to control vascular patterning independent of NRP [98]. Nevertheless, the extracellular domain of NRP1 can modulate SEMA3E-triggered plexinD1 signaling [99]. In addition to lateral interactions, transcellular interactions are also possible for NRP. Tumor-penetrating peptides interact with the arginine-binding pocket within the b1 domain of NRP. The snake-venom-derived rhodocetin-αβ also interacts with the b1 domain and recruits NRP1 to the hepatocyte growth factor (HGF) receptor, MET. a1/2 domain, grey diamonds; b1/2 domain, light grey ellipses; c domain, black ellipse.

NRPs are versatile in their structure and in their repertoire of soluble ligands which they bind, as well as in their ability to form holoreceptors with different coreceptor molecules to modulate important cell functions (Figure 2) [95]. These diverse combinations have different affinities for various ligands, e.g., Nrp1 homodimers preferentially bind SEMA3A, while NRP2 homodimers recognize SEMA3F [96]. Moreover, after ligand binding, they can form supramolecular protein complexes in the cell membrane [97]. Due to their modular structure, NRPs present binding sites for various ligands, which therefore need not necessarily compete for binding to NRP. By recruitment of receptor kinases, NRPs act as coreceptors despite lacking an intracellular kinase domain and confer stimulatory or inhibitory signals, depending on the soluble ligand and the receptor kinase recruited.

### 4.1. NRP1 Interaction with VEGFs and VEGFRs

Among the ligands of NRP1-containing signal complexes, VEGF-A is the best-understood binding partner of NRP1 and forms the molecular basis for the development of pharmaceutical compounds that affect VEGF-A binding to the NRP1/VEGFR2 complex [100,101]. Such pharmacological compounds may help to inhibit tumor angiogenesis in tumors and to treat other diseases in which angiogenesis is a key feature, such as age-related macular degeneration, rheumatoid arthritis, psoriasis, diabetes-induced ocular neovascularization, inflammatory diseases, ischaemia/reperfusion injury, infantile haemangioma, and atherosclerosis [102].

VEGF-A, encoded in a gene locus on chromosome 6p21.1, occurs in six different splice variants [103]. All VEGF-A isoforms comprise amino acid sequences which are encoded by the first five exons and contain the relevant residues for binding to the VEGF receptors, VEGFR1 and VEGFR2 [102]. However, not all VEGF-A splice variants can tether to glycosaminoglycan chains of extracellular matrix (ECM) proteins or bind to NRP1, as the relevant residues are located in exons 6 and 7, and in exons 7 and 8a, respectively [102]. Hence, NRP1 binds the VEGF-A splice variants VEGF-A_165_ and VEGF-A_189_, but not the smaller variants with 145, 121, or 120 amino acids in length [13,104,105,106,107,108]. Remarkably, NRP2 binds VEGF-A_145_ in addition to VEGF-A_165_, but not VEGF-A_121_ [109]. Two VEGF-A molecules form a dimer via two interchain disulfide bonds [110], which can then bind to the homodimeric VEGF-A receptors VEGFR1 and VEGFR2 in complex with a NRP1 homodimer. Thus, a ternary VEGF-A/VEGFR/NRP1 complex is formed with a putative 2:2:2 stoichiometry [102,111]. Four non-contiguous amino acids, two arginine and two glutamate residues within the C-terminal part of the VEGF-A molecule are responsible for binding to VEGFR with high affinity in the nanomolar range [100,101,102,112]. The C-terminal arginine residue of VEGF-A_165_ is of particular importance as it binds to a binding pocket within the NRP1-b1 domain formed by the side chains of residues Y297, Y353, D320, S346 [100,101]. Several other NRP1 ligands also possess such a C-terminal arginine residue, leading to the formulation of the carboxy-terminal end rule (CendR), which states that NRP1 recognizes peptides with a C-terminal arginine residue with its binding pocket in its b1 domain [40].

The 165 amino acid long variant of the vascular endothelial growth factor (VEGF-A_165_) was first identified to be a ligand for NRP1 [13]. Unlike its shorter splice variant VEGF-A_121_, dimeric VEGF-A_165_, binds with its C-termini to the NRP1 tandem domain b1/b2 and concurrently to the ectodomain of the VEGF receptor-2 (VEGFR2) [13,104,105,106,107]. As a result, dimeric NRP1 and dimeric VEGFR2 receptor come into close contact, thus allowing a mutual interaction of all partner proteins within a ternary VEGF-A_165_/VEGFR2/NRP1 signaling complex [113]. This complex is predominantly found in arterial ECs where it promotes angiogenic sprouting [114]. Although VEGF-A_121_ can also bind directly to NRP1, it is incapable to induce the formation of a NRP1/VEGFR2 holoreceptor [115].

The repertoire of NRP2 ligands within the VEGF family comprises VEGF-A_145_ and VEGF-C. Upon binding its ligand, NRP2 forms a ternary complex with VEGF-C and VEGFR3 on lymphatic ECs and is involved in lymphangiogenesis [61,116].

### 4.2. NRP1 Interaction with Other Growth Factor Receptors

Recently, other soluble ligands and other receptor partners of NRP1 have been reported, demonstrating an amazing versatility of NRP1 in this respect. Interestingly, by binding to several growth factors other than VEGF-A, NRP1 becomes a coreceptor for the respective growth factor receptor, thus promoting the formation of a ternary signal complex. In such a way, the following growth factors and receptors may interact with NRP1: Placenta growth factor (PlGF), which is a member of the VEGF protein family and its receptor PlGFR [117,118], hepatocyte growth factor/scatter factor (HGF/SF) and its receptor MET [119,120], fibroblast growth factor-2 (FGF-2, also known as basic fibroblast growth factor, bFGF) [121], keratinocyte growth factor (KGF) [122], platelet-derived growth factors C and D (PDGF-C and PDGF-D) [77,123,124], as well as transforming growth factor-β (TGF-β) [125,126,127] and their respective receptors. Although NRP1 itself does not bind epidermal growth factor (EGF), its extracellular domain nevertheless regulates ligand-engaged EGFR oligomerization and endocytosis [3].

With the exception of the receptor-type serine kinases TGF-βRI and TGF-βRII that activate SMAD2 and SMAD3, all NRP1-associated growth factor receptors are receptor-type tyrosine kinases that autophosphorylate upon agonistic stimulation and recruit adapter proteins to trigger an intracellular signaling cascade [127]. As part of this holoreceptor complex, NRP1 takes the role of a matchmaker for growth factors and their cognate receptors and influences their interaction. The functional SEMA3A receptor in ECs, for example, consists of a tripartite complex of NRP1 with plexinA1 and plexinD1 [128].

### 4.3. Interaction of NRPs with Semaphorins and Plexins

Furthermore, several members of the semaphorin family also bind to NRPs [95,129]. As physiological mediators of antiangiogenic cues, they play an inhibitory role in tumor angiogenesis and tumor growth in addition to their involvement in neuronal development as soluble chemorepellents [130].

The seven class 3 semaphorins, SEMA3A-G, are secreted members of a large family of guidance factors that regulate processes, such as developmental and tumor angiogenesis, by signaling through receptors composed of NRP1 or NRP2 and plexinA or plexinD, respectively [63]. ECs express all four A-plexins to varying degrees, with plexinD1 being most strongly expressed [98,128]. SEMA3A binds to NRP1-containing holoreceptors, and SEMA3F binds to NRP2-containing holoreceptors, thereby promoting normalization of the cancer vasculature and inhibiting metastasis [131,132]. In contrast, SEMA3C binds to both NRP1 and NRP2 with similar affinity [133].

Semaphorin SEMA3A binds to the a1/a2 tandem domain of NRP1 and causes collapse and retraction of the nerve growth cone [134]. The interaction of NRP with plexin is mainly mediated by motifs within the transmembrane domains [135]. In addition, juxtamembrane regions of NRP, including the MAM domain, also contribute to the contact surface with partner receptors. [16]. SEMA3A binds as a homodimeric molecule to the dimeric NRP1/plexinA1 complex in a 2:2:2 stoichiometry [136]. In this complex, the interchain contact surface of an unbound SEMA3A dimer that is formed by the top face of its 7-blade β-propeller domain (SEMA domain) is disrupted. Instead of the homophilic interaction within the SEMA3A-dimer, the SEMA3A dimer opens and allows access of the NRP a1/a2 tandem domain to the top face of its SEMA domains [136]. In consequence, the two plexinA1 molecules, which also interact via their SEMA domains with the more membrane-proximal domains of the NRP1 dimer, interact with the SEMA domains of NRP1-bound SEMA3A and undergo a conformational change. As a result, autoinhibitory contact sites in plexinA1 are exposed. Thus activated, they trigger an intracellular signal, which eventually leads to growth cone collapse in neurons [137]. In this model, NRP1 not only serves as matchmaker between semaphorin ligand and plexin receptor, but also acts as helper protein that assists in rearranging the homophilic SEMA-domain interaction within the SEMA3A dimer into a heterophilic interaction between the SEMA domains of SEMA3A and plexinA1.

NRP2 has been originally described as receptor for SEMA3F (at that time referred to as sema IV) that mediates its repulsive effect on growing neurons and is able to form heterooligomers with NRP1 [97]. In comparison to NRP1, the repertoire of NRP2 ligands within the semaphorin family is more limited and, in addition to SEMA3F, also includes SEMA3G, while the semaphorins SEMA3B, SEMA3C, and SEMA3D can interact with both NRP1 and NRP2 [138].

### 4.4. NRP1 Interaction with Integrins

Integrins have also been reported to interact with NRPs [114,139,140,141,142]. They are a family of cell adhesion molecules that consist of two subunits, α and β, both of which span the cell membrane with an α-helical transmembrane-domain [143,144]. The two integrin subunits form an extracellular head domain harboring the binding site for ECM ligands [145,146,147]. The head domain is connected via two stalks that are formed by each chain to a transmembrane domain [143,144]. Upon ligand binding to the head domain, integrins undergo dramatic conformation changes, thereby transducing signals between the ECM and the cell [144,148]. Similar to NRPs, integrins lack an intracellular kinase domain. Ligand binding induces integrin clustering into supramolecular complexes, termed adhesomes [149,150]. Consequently, the cytoplasmic domains of the integrins recruit adapter proteins and signaling molecules for cytoskeletal attachment and signal transduction, respectively [148,151]. Various possibilities for the interaction of integrins with NRPs have been discussed: An intercellular interaction between integrins α5β1 and α9β1 on cancer cells with NRP2 as counterreceptor on ECs enhances tumor cell spreading and metastasis [87,152]. Other reports describe a lateral interaction of integrin α6β1 with NRP2 on cancer cells and of integrin α5β1 with a VEGFR2-NRP1 complex on ECs [114,139,140,142,153]. The latter even enhances the α5β1 integrin-mediated remodeling of the fibronectin matrix [141]. However, it is not yet clear whether integrins can come into direct physical contact with NRP1, similar to the complex formation of NRPs with receptor kinases [114,153]. Irrespective of such a potential direct contact, NRPs are found in integrin-containing multi-protein complexes of adhesomes [142,153]. This explains the regulatory interaction between integrins and NRPs, for example in the upregulation of the collagen-binding integrin α2β1 upon stimulation with the NRP1-agonist SEMA3A in breast cancer cells [154], the enhanced expression of αvβ3 integrin upon blockage of NRP1 [155], and reciprocally the inhibitory sequestering of NRP1 from the NRP1-VEGFR2 signaling complex by αvβ3 integrin in ECs [156]. Moreover, NRP1 influences the binding response of integrin αvβ3 to tenascin C, an adhesion-modulating ECM-protein, in breast cancer cells [74].

### 4.5. NRP1 Interaction with Other Molecules

Another coreceptor of NRP1 is the L1 cell adhesion molecule (L1CAM) which belongs to the immunoglobulin superfamily (IgSF), mediates intercellular contacts between neurons, and interacts with NRPs in both cis- and trans-cellular manner [157,158]. The α-helical transmembrane domains of L1CAM and NRPs likely mediate the physical interaction between both proteins [132]. Association of NRP1 with L1CAM triggers disassembly adhesomes in growth cones and subsequent growth cone collapse by recruitment and activation of the FAK/MAPK signal cascade [159]. Likewise, on neurons, NRPs interact with the p75 neurotrophin receptor and thus regulate apoptosis of nerve cells and inhibition of myelin growth [160]. 

Heparin and heparan sulfate have been described as binding partners of neuropilins [13]. Heparin of at least eight monosaccharide units can directly bind to NRP1, and it significantly enhances binding of VEGF-A_165_ and PlGF-2, to the b1b2 domain of NRP1 when its chain length is at least 20–24 monosaccharides [118]. By physically interacting with both ligand and receptor, it is an important regulator of VEGF-A_165_ and PlGF-2 interaction with NRP1 on ECs [118]. As NRP1 possesses a “heparin” mimetic site that can interact with the heparin-binding site of diverse proteins, such as FGF-2 and HGF, NRP1 is able to regulate the activity of these heparin-binding proteins [121]. Heparan sulfate, rather than heparin, is the natural cell surface polysaccharide in vivo. Thus, heparin binding VEGF-A isoforms, for example, which are differentially sequestered by heparan sulfate proteoglycans in the ECM, can be released from the ECM and bind to heparan sulfate proteoglycans on the EC surface to regulate NRP1 and VEGFR signaling and angiogenesis [161,162,163,164,165].

NRP1 is also a target of soluble toxins, such as the αβ subunit of rhodocetin, a venom component of the Malayan pit viper (*Calloselasma rhodostoma*). Rhodocetin αβ (RCαβ) has been identified as the first non-enzymatic component of a snake venom that recognizes NRP1 on ECs [166]. This C-type lectin-related protein binds to the b1/b2 tandem domain of NRP1 and induces the formation of a ternary complex with MET on endothelial and tumor cell membranes [166]. Like the physiological MET ligand, HGF, rhodocetin-αβ thereby alters adhesomes and increases cell motility [166,167].

### 4.6. NRP1 Can Trans-Interact with Ligands on Neighboring Cells

While most of the interactions between NRPs and their coreceptors occur via lateral contacts in the plasma membrane of the same cell, several reports also describe a trans-interaction of NRPs on one cell with a co-receptor on an adjacent cell [68]. Such transcellular interactions occur between EC-anchored VEGFR2 and NRP1 of tumor cells [68,168]. Likewise, they have been described for the immune synapse with its close interaction between antigen-presenting dendritic cells and T_reg_ cells expressing membrane-bound SEMA4A and NRP1, respectively [169,170]. Remarkably, NRP1 can be transferred from ECs to T lymphocytes by the formation of an immune synapse, whereupon the T cells start to express VEGF-A_165_, which in turn amplifies signaling via NRP1/VEGFR2 trans-interaction in ECs during inflammation [171].

## 5. Signaling and (Patho) Physiological Functions of NRP

**Figure 3:**
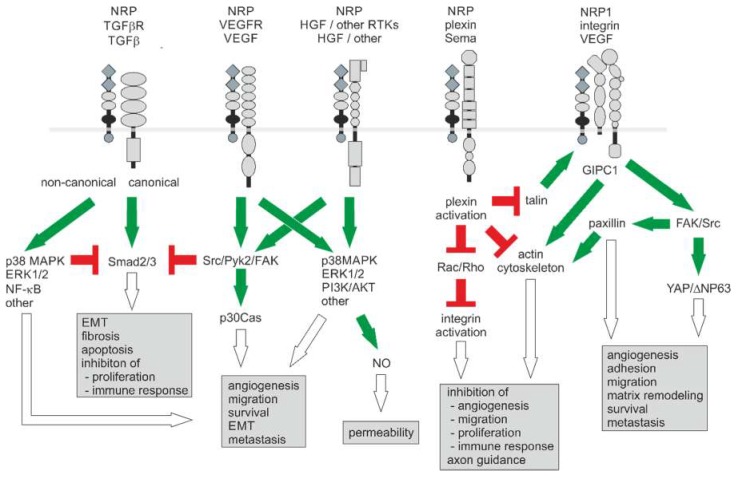
Neuropilin-1-triggered signaling pathways in the tumor vasculature and the tumor microenvironment. With two NRPs, numerous receptor/coreceptor combinations are possible, which can trigger a large number of cellular responses. For clarity, only monomers of receptors and coreceptors are shown. For signaling, dimerized ligands bind to a NRP dimer that interacts with a pair of receptor type kinases or plexin receptors. Activating and inhibitory signals are indicated in green and red, respectively. The resulting effects are indicated by open arrows. Modified from [174].

NRPs are multifunctional non-tyrosine kinase receptors for VEGF, TGFβ, and semaphorins, which are, in addition to their role in axonal guidance, associated with tumor proliferation, angiogenesis, and survival by triggering growth-promoting signal transduction pathways (Figure 3) [90,158,159]. With their extracellular domains, NRPs may act as matchmakers and effectors for the binding of various growth factor receptors with their respective ligands as they influence specificity and affinities of the NRP-containing holoreceptor. Moreover, they may assist in presenting interaction sites between ligand and receptors, such as the SEMA contact face between SEMA3A ligand and plexin A1. Another way of causing an effect is that NRP1 can regulate trafficking of VEGFR or can sequester VEGF-A ligand by its ability to interact with its partner proteins via its extracellular domains [172,173]. In contrast, the cytoplasmic domain of NRP1 does not seem to be crucial in triggering an NRP1-autonomous signal transduction, as a knock-in mouse model expressing cytoplasmic domain-truncated NRP1 shows only minor vascular defects as opposed to the lethal phenotype and several vascular malformations in the global knockout of the entire NRP1 molecule.

### 5.1. NRP Modulates Receptor Tyrosine Kinase Signaling

As a coreceptor of VEGF receptors -1 and -2, NRP1 modulates VEGF signaling, and in a VEGF-receptor-independent manner as a coreceptor of plexin-A, it mediates the chemorepulsant activity of semaphorins [13,175]. Cancer cells of solid tumors express varying levels of VEGFR1, but hardly VEGFR2 and -3, which is why they probably bind VEGF-A mainly via NRP1 [172,173,176,177]. It is still unclear whether there is VEGFR-independent NRP1/VEGF-A signaling in ECs [163,164]. In the trimeric NRP1/VEGFR2/VEGF-A complex, signaling likely occurs via mutual phosphorylation of the cytoplasmic tails of the two VEGFR2 receptor molecules within the complex, which activates the kinase domains and leads to activating phosphorylation of downstream signaling molecules. Signal transduction involves the two major activation axes of PI3-kinase, including Protein kinase B (AKT), and of PLCγ, including RAS-RAF-ERK [114,164].

In contrast, cells from skin cancer, prostate cancer, and glioblastoma cells largely lack VEGF receptors -1 and -2. Consequently, VEGF-A-induced RhoA activation depends mainly on NRP1 [176]. Yoshida and coworkers demonstrated that binding of VEGF-A to NRP1 causes the interaction of NRP1 with the scaffold protein GIPC1. This promotes the formation of a molecular complex of GIPC1 and Syx, a guanine nucleotide exchange factor (GEF) for RhoA, leading to an increase in the GTP-bound active form of RhoA [176,178]. In ECs, on the other hand, NRP1-mediated RhoA activation stimulates cell motility via the PI3K pathway [179]. VEGF-A in the tumor microenvironment affects RhoGEF expression of ECs [180]. Increased levels of active RhoA and ROCK in tumor ECs, due to a disturbed perception of mechanical forces between them and their surrounding ECM, contribute to impaired vascularization [181]. Therefore, intervention in the VEGF-A-RhoA signaling pathway could be promising for cancer therapy [178]. For VEGF-C-induced activation of AKT, VEGFR3/VEGFR2/NRP1 must be formed, whereas ERK1/2 is activated mainly NRP-independently [182].

In tumor cells, EGF induces expression of NRP1 [183,184,185]. In turn, NRP1 can complex with EGFR, which is overexpressed and active in many cancer cells. EGFR, also known as Her2 and ErbB2 (Erythroblasotsis oncogene B), is a RTK of the EGFR family that can form holoreceptors with EGFR, ErbB3, and ErbB4 [186]. Remarkably, the ectodomain of NRP1 is capable to selectively trigger phosphorylation of EGFR without interfering with EGF-induced receptor activation [3]. EGF and TGF-β, respectively, induce NRP1-dependent EGFR clustering and endocytosis resulting in AKT signaling [3]. The endocytosis of plasma membrane-bound EGFR is controlled independently from tyrosine autophosphorylation by NRP1-mediated receptor oligomerization and clustering [3]. Anti-EGFR therapy in the context of cancer treatment may have cardiotoxic side effects, maybe due to EC dysfunction, because EGFR, which is normally involved in EGF/neuregulin signaling, can also form a holoreceptor with NRP1 and elicits a repellent SEMA3D signal in venous ECs [187]. 

NRP2 also regulates the number of EGFR on the surface of cancer cells, thereby controlling EGF-mediated signaling and response to EGFR-targeted therapy [188]. Increased expression of NRP2 results in down-regulation of EGFR, slowed tumor growth, and suppression of an EGFR “rescue” pathway of tumor cells, which is turned on as a protective response to MET-directed tumor therapy [188]. Accordingly, the development of resistance to MET-directed therapy is associated with a loss of NRP2 and thus an activation of NFκB signaling [188]. At the same time, the EGFR-associated protein Cell migration inducing hyaluronidase 1 (CEMIP, KIAA1199), which counteracts the degradation of activated EGFR kinase, is upregulated [188].

The role of NRP1 as a coreceptor for FGFs in (tumor) vasculature is still unclear, as NRP1 may bind several FGFs [121] but does not affect the FGF-2-induced proliferation of HUVECs [96]. Apparently, SEMA3A inhibits FGF-2-induced proliferation-promoting ERK1/2 activation in ECs downstream of the RTKs [96].

The interaction of NRP1 with PDGF-A and -B might be relevant for EMT [172,189]. Stimulated by PDGF, as well as by VEGF and HGF, NRP1 promotes phosphorylation of p130Cas via its cytoplasmic domain and, thus, cell motility, independently of GIPC1, [31,189,190,191,192,193]. PDGF-D can bind to NRP1 and induce the formation of a holoreceptor with PDGFRβ, thereby stimulating proliferation in fibrotic processes and various cancers [179,194,195,196]. By PDGF-D, NRP1 is displaced independently of PDGFRβ into intercellular junctions, thus reducing VEGFR2 signaling [124]. The interaction between NRP1 and PDGFRβ induced by PDGF-D can also occur in trans between ECs and PCs [124].

NRP1 and NRP2 can also interact as coreceptors for HGF with the hepatocyte growth factor receptor/scatter factor receptor MET on ECs [120,197]. HGF stimulates, via NRP1 tyrosine phosphorylation of p130Cas, EC motility and proliferation [193]. In carcinoma cells, NRP1 is essential for activation of tumor growth- and invasiveness-promoting pathways involving p38MAPK, Src, and PI3K and for NRP1/Met-complex internalization [119].

Often, oncogenes switch signal pathways in such a way that they become essential regulators of proliferation and survival of tumor cells, termed oncogene addiction [198]. Downregulation of NRP2 in MET-addicted cells leads, by compensatory enhancement of EGFR signaling, to EGFR-dependent resistance to targeted therapies [188]. As NRP1 is a promiscuous coreceptor for different growth factor receptors, its depletion or inactivation may inhibit various signaling cascades starting from VEGFR2, EGFR, and MET, and thus, may aid to curb cell proliferation and tumor angiogenesis and oncogene addiction. This strategy seems feasible and is reinforced by the following observations. In a xenograft mouse model of gastric cancer, NRP1-depletion causes upregulation of p27 and downregulation of cyclin E and Cyclin-dependent kinase-2 (CDK-2) and, thus, cell cycle arrest in the G1/S phase [199]. Also along this line, downregulation of NRP1 counteracts the adverse effects of acquired resistance to EGFR, MET, and BRAF (Rat/rapidly accelerated fibrosarcoma, isoform B) inhibitors, as NRP1 upregulates alternative tumor-promoting effector kinases EGFR and IGF1R (insulin-like growth factor 1 receptor), respectively, via a c-Jun N-terminal kinase (JNK)-dependent signaling cascade [200].

### 5.2. NRP Modulates TGF-β Receptor Signaling

NRP1 can function as a coreceptor for TGFβ by forming holoreceptors with TGFβ-receptors I, II, and III [126,201]. By forming a holoreceptor for TGF-β, NRP1/TGF-βR can control angiogenic sprouting independent of VEGFR2 [202,203].

NRP1 has a negatively charged cleft in its b1 domain which allows binding of various ligands, such as TGF-β [28,125]. NRP1 on breast cancer cells binds both latent and active TGF-β1 with high affinity [125]. Binding of TGF-β by NRP1 promotes a myofibroblast phenotype [125,201]. Downregulation of NRP1 in stromal fibroblasts reduces TGF-β-induced SMAD2/3 phosphorylation and thus expression of α-smooth muscle actin [126,201]. As recently reviewed, TGF-β and Ras signaling converge and feed back onto the expression of NRP1 [127]. Reduced expression of NRP1 in KRAS-transformed cells results in reduced SMAD2 phosphorylation and increased tumor growth [127,204]. In breast cancer cells, TGFβ levels as well as the downstream expression of NRP1 and SMAD-2 are negatively regulated by microRNA (miR)-206, and overexpression of miR-206 inhibits EMT as well as migration and invasion of breast cancer cells [205]. TGFβ1-mediated inhibition of miR-196a-3p and consequent activation of NRP2 promotes a metastatic phenotype of breast cancer cells [206].

### 5.3. NRP Modulates Semaphorin/Plexin Signaling

The binding and signaling of VEGF and related growth factors seem to be antagonized by class 3 semaphorins [16]. Additionally, soluble NRP isoforms modulate these signaling processes [16].

Although NRP1 is the specific SEMA3A ligand, NRP1, with its short intracellular domain, cannot transduce SEMA3A signals on its own, but relies on complex formation with plexinA receptors [207]. By binding of Sema3, small GTPases, such as R-Ras, are inactivated by the intracellular plexin domain, which then promote integrin-mediated cell-matrix interaction [63,208].

By binding to NRP1 and plexinD1 in ECs, SEMA3C induces internalization of VE-cadherin and shutdown of VEGF-induced signaling via AKT, FAK, as well as p38MAPK, which then causes disassembly of EC junctions and focal adhesions and related cytoskeletal rearrangement [209]. Thus, SEMA3C signaling can induce EC apoptosis and inhibit pathological angiogenesis [209]. In cancer, SEMA3C and its receptors are often highly expressed and associated with invasion and metastasis [210].

SEMA3F overexpressing melanoma cells form poorly vascularized tumors because Nrp2 inhibits tumor development and metastasis by a strong antiangiogenic cascade [211,212]. In ECs of premalignant lesions, SEMA3A is an endogenous angiogenesis inhibitor whose expression is lost during tumor progression [133]. NRP2 signaling in pancreatic adenocarcinoma promotes tumor angiogenesis by increasing Jagged1 levels [86]. Overexpression of Jagged1 in cancer cells promotes neovascularization and growth of experimental tumors in mice [213]. Jagged1 is an important regulator of tip cell formation in the angiogenic endothelium because of its ability to modulate Delta-like 4 (Dll4)-Notch signaling [214]. 

Recruitment of pericytes to nascent vessels is essential for development, stabilization and maturation of the vasculature [215,216,217], and it depends, inter alia, on SEMA3A/NRP1 signaling [218,219]. In tumor angiogenesis in vitro, invasive cancer cells recruit less pericytes than non-invasive cancer cells [192]. NRP1 is involved as a PDGF-B coreceptor in the differentiation of pericytes from mesenchymal stem cells [192]. Compared to normal blood vessels, the tumor vasculature has comparatively few pericytes, which is one reason for their leakiness [220]. Expression of SEMA3A normalizes pericyte coverage and at the same time reduces angiogenesis and tumor growth [218,221].

### 5.4. NRP Modulates Signaling of Integrins

NRPs interact with specific integrins and activate them to bind to ECM molecules [139,156,222]. Integrins also lack a signaling domain but associate within adhesomes with various kinases, such as the Src-family members and focal adhesion kinase (FAK) [223]. NRP1, as a VEGFR2 coreceptor, can modulate the PI3K/AKT/PTEN signaling axis that activates the inside out signaling of β1, β3, and β5 integrins [224]. Both NRP1 and NRP2 can directly associate with β1 integrins [90,142,225]. NRP modulates specific integrins contributing to tumor initiation and progression, such as α2β1, α5β1, and β3 [153]. In SEMA3A-expressing breast cancer cells, an autocrine feedback loop activates the serine/threonine kinase GSK-3 via NRP1 and thus the expression of integrin α2β1, thereby attenuating migration and invasion [154]. Similarly, inhibitory autocrine SEMA3 loops fine-tune integrin α5β1- and αvβ3-mediated adhesion to the ECM to give ECs the necessary flexibility and mobility during angiogenesis [225]. In ECs, the interaction between NRP1 and a5β1 is mediated by GIPC1, which links these two receptors by binding to PDZ binding domain sequences in their cytoplasmic tails [139]. During cell migration, SEMA3A binds to a NRP1/plexinD1 holoreceptor in integrin-containing focal complexes at dynamic cell protrusions and there modulates the activity of integrin α5 [139,225]. Here, the intracellular GTPase activation protein (GAP) domain of plexin activates the small GTPase Rap1 (Ras-related protein), which promotes via effector proteins, such as RIAM1, conformational activation of integrins through talin [226]. Angiogenesis can be regulated by complex formation of β3 integrins with NRP1, thereby reducing the number of NRP1/VEGFR2 holoreceptors available for VEGF-A signaling [156].

In a similar way, NRP2-mediated VEGF signaling in breast cancer and prostate carcinoma cells is subject to modulation by integrin α6β1 [90,227]. NRP2/VEGF signaling activates the TORC2/PKC pathway that activates integrin a6β1 and promotes its association with F-actin, possibly through phosphorylation of the integrin α6 subunit [227,228,229]. This then triggers formation of focal adhesions and hence allows focal adhesion signaling [227]. In breast carcinoma cells, NRP2/VEGF signaling increases FAK indirectly by activation of integrin α6β1 [227]. NRP1 can also form a ternary complex with GIPC1 and integrin α6β4 [226]. Via this complex, VEGF-A can trigger FAK/Src signaling in epidermal cancer stem cells. This stabilizes YAP1/ΔNP63α and thus enhances survival, invasiveness and tumor angiogenesis [226].

### 5.5. NRP Modulates Signaling of Other Extracellular Ligands

Galectins are β-galactoside-binding proteins with mostly angiostimulatory activity, possibly by modulating receptor endocytosis [230,231]. In contrast to the VEGFR-binding galectin 3, NRP1 directly binds galectin 1, which is overexpressed in tumor-associated capillary ECs in squamous cell carcinoma [232]. This binding increases proliferation and adhesion of ECs by enhancing the phosphorylation of VEGFR2 and triggering signaling via MAP kinases SAPK1 and Jnk. In combination with VEGF, cell migration is additionally increased [232].

Binding of the snake venom component, rhodocetin αβ (RCαβ) to NRP1 induces the formation of a ternary complex with MET on endothelial and tumor cell membranes [166]. This leads to Y1234/1235 phosphorylation of MET and, via subsequent paxillin phosphorylation at Y31, causes a rearrangement of cell-matrix anchoring complexes of focal adhesions into focal contacts and reorganization of the actin cytoskeleton, thereby reducing cell adhesiveness and increasing cell motility [166,167]. In in vivo tumor models, RCαβ selectively destroys blood vessels of tumor tissues, but not of normal tissues, by eliciting reactions initially of the tumor cells and subsequently on ECs of the tumor microenvironment [167].

### 5.6. Effects of Intracellular Partners and PDZ-Binding Proteins on NRP Signaling

Upon binding of VEGF, NRP1 triggers RAS activation and consecutively phosphorylation of ERK1/2 and AKT [233]. Notably, wild-type KRAS is found in tumors where NRP1 has tumor-promoting properties, while in tumors where NRP1 acts as a tumor suppressor, oncogenic KRAS mutations are found [52]. Oncogenic KRAS and TGF-β signaling induces the major transcription factor Snail, which down-regulates transcription of E-cadherin [127,234]. Moreover, in cells with oncogenic but not wild-type KRAS, TGF-β downregulates NRP1 at both transcriptional and translational level [204]. Such downregulation of NRP1 in oncogenically KRAS-transformed cells promotes tumor growth by reducing SMAD2 phosphorylation [204].

Binding of GIPC1 to the cytoplasmic SEA (Ser-Glu-Ala) motif of NRP stimulates internalization of integrin α5β1 in Rab5-positive early endosomes [139]. Moreover, this GIPC1- integrin α5β1 complex also interacts with the myosin VI motor protein thereby promoting EC adhesion to fibronectin and integrin a5β1 endocytosis [139].

### 5.7. NRP Signaling is Regulated by Endocytosis

Membrane trafficking also plays a key role in regulating signaling pathways [235]. Endocytosis is an essential process in NRP signaling not only in neural cells, which internalize NRP1/plexin holoreceptors with bound SEMA3A in association with L1-CAM by endocytosis [158]. In ECs, NRP1-guided endosomal translocation of VEGFR2 significantly influences VEGF-A-induced ERK1/2 activation [236,237]. In addition, activation of p38 MAPK depends on endosomal signaling of NRP1 [238]. In interacting adjacent cells, endocytosis of VEGF-A-VEGFR2 complexes is prevented by incorporation of trans-standing NRP1 into holoreceptor complexes, thereby regulating angiogenesis, tumor initiation and tumor angiogenesis [68,168].

NRP1 and NRP2 can form docking sites for the endocytic adapter protein GIPC1 (also known as NIP, SEMCAP1, synectin, IIP1, TIP2, and GLUT1CBP) [16,239,240]. In complex with GIPC1, NRP1 can regulate trafficking and recycling of clathrin-coated vesicles [241]. In this way, NRP1 is involved in nutrient uptake by tumor cells, and its surface expression inversely correlates with nutrient supply [242]. NRP1-mediated endocytosis (micropinocytosis and a related, yet different process [242]) allows tumor cells as well as other cells to take up nutrients. Moreover, it is relevant for the introduction of drugs into the tumor cells. Interestingly, these do not have to be covalently coupled to tumor targeting molecules but can be taken up collaterally [242,243].

### 5.8. NRP Regulates Hedgehog and Wnt/β-Catenin Pathways

NRPs can activate signaling pathways that protect cancer cells from cytotoxic drugs and apoptosis. NRP1 and NRP2 are major positive regulators of the Hedgehog (HH) signaling pathway, which is relevant to angiogenesis and wound healing. It promotes EMT, cancer stem cell (CSC) survival, and tumor growth [172,244,245]. NRP-triggered HH signaling modulates the activity of other signaling pathways, such as Wnt/β-catenin, Notch, and TGF-β [159]. In a positive feedback loop, NRP1 transcription is induced by HH signaling, which in turn increases HH target gene activation [245]. SEMA3 enhances the direct binding of phosphodiesterase 4B (PDE4D) to NRP and thus promotes the hydrolysis of cAMP at the plasma membrane, which inhibits protein kinase A (PKA) and controls HH signaling [246]. GLI1, a downstream effector of the HH pathway, is non-canonically activated by MAPK/ERK in the majority of lung adenocarcinomas, and especially in their CSC compartment, while the canonical pathway activator, Smoothened, is only weakly expressed [247]. The MAPK/ERK/GLI1 signaling cascade apparently is triggered by KRAS mutation and stimulation of NRP2 by VEGF. VEGF is potentially provided by CSCs or stromal cells in an autocrine or paracrine manner, respectively [247]. GLI1 in turn induces expression of BMI-1, a key stem cell factor, in breast cancer and enhances expression of integrin α6β1 and NRP2 in an autocrine loop [142].

In medulloblastoma, tumor derived Sonic hedgehog (SHH) induces PlGF production in the cerebellar stroma, which promotes tumor cell survival through NRP1 independent of VEGFR1 [248]. The majority of medulloblastomas with constitutively active Wnt signaling does not express NRP1 due to a high expression of miR-148a, which downregulates NRP1 by binding to the 3′ untranslated region (3′-UTR) of its mRNA [249].

In contrast, NRP1 expression is induced by Wnt/β-catenin signaling in mammary stem cells and in mouse mammary tumor virus (MMTV)-Wnt1 tumor xenografts [250]. In breast cancer, NRP1/VEGF-A signaling promotes a CSC phenotype and the formation of aggressive and highly vascularized tumors by activating the Wnt/β-catenin pathway [174]. Likewise, vascular progenitor cells depend in their NRP1 expression on Wnt and BMP4 signaling, intercellular contact, hypoxia, and hemodynamic stimulus [251]. Also, in biliary tract cancer with strong expression of NRP1 and NRP2, Wnt and PI3K signaling are associated with tumor angiogenesis [252]. Moreover, HGF, secreted by myofibroblasts, induces Wnt signaling in colorectal CSCs and thus contributes to the maintenance of their stemness [253].

NRP2 has been implicated in metastatic progression, although it usually occurs in carcinomas just in small amounts [2]. NRP2 occurs in gastric cancer, and its silencing in a gastric cancer cell line leads to a decreased expression of the metastasis mediator S100A4, mediated via Wnt/β-catenin signaling and accompanied by downregulation of anti-apoptotic B-cell lymphoma 2 (Bcl-2) and concomitant upregulation of pro-apototic caspases -3 and -7 [254]. Presumably, NRP2 promotes TGF-β1 or β-catenin/Wnt signaling in response to paracrine VEGF [142,255]. In osteosarcoma cells, NRP2 expression is important for the recruitment of HUVECs, and its expression can be downregulated by overexpression of Wnt signaling antagonists, such as soluble LRP5, Frzb, and WIF1 [256].

### 5.9. Soluble NRPs Act as Decoy Receptors

The binding of VEGF and related growth factors to NRPs and corresponding signaling seem to be antagonized by class 3 semaphorins [16]. Additionally, soluble NRP isoforms modulate these signaling processes [16]. Secreted sNRP1 lacking a MAM dimerization motif functions as VEGF antagonist by scavenging VEGF-A_165_ and inhibiting its interaction with membrane-bound receptors [22]. Accordingly, sNRP1 inhibits tumor angiogenesis and tumor progression [23]. Artificially dimerized sNRP1, in contrast, delivers VEGF-A_165_ to VEGFR2 expressing ECs promoting angiogenesis [24].

A soluble splice variant of NRP2, s9NRP2, scavenges VEGF-C and inhibits VEGF-C/NRP2 signaling in prostate cancer. This, suggests that s9NRP2 may be used therapeutically in the treatment of tumors that strongly depend on the VEGF-C/NRP2 axis for survival [257].

### 5.10. Regulation of NRP Expression as a Potential Feedback Loop of NRP Signaling

Expression of NRP1 is induced by growth factors via the RAS/MAPK signal pathway [184,250,258]. A de novo expression of NRP1 in BRAF-addicted melanoma cells contributes significantly to their development of secondary drug resistance by adapting their gene expression, such as upregulation of EGFR [200,259,260].

NRP1 is instrumental in microRNA-based intercellular communication, because it efficiently binds Argonaute2 (AGO2) and AGO2/miR complexes without any involvement of its VEGF binding site, and it promotes their internalization into cells, whereupon they can perform their function and promote, for example, proliferation, migration and angiogenic tube formation [261]. MicroRNAs (miRs) are small, non-coding RNA molecules that posttranscriptionally regulate the expression of most genes by specifically binding to the 3’-UTR of a mRNA they regulate, thereby either inhibiting their translation or initiating their degradation [262]. miRs are important for intercellular communication and significantly affect the development and progression of many malignancies. Long non-coding RNAs (lncRNAs) interact, among others, with miRs. Therefore, they play a major role in all stages of tumorigenesis and metastasis by [263]. In colorectal cancer, miR-206 inhibits tumor growth and invasion by downregulating the long non-coding RNA lnc00152 which again promotes NRP1 expression and EMT [264]. In addition, miRs can also mutually regulate their activity. In colorectal cancer metastasis, for example, miR320b abolishes downregulation of NRP1, β-catenin, and Rac-1 by competing with miR320a [265].

The transcription factor SOX10 induces among others the expression of miR338-p, which in gastric cancer and oral squamous carcinoma, inhibits the expression of NRP1 via phosphorylation of ERK1/2, MAPK, and AKT [266,267]. While transcription of NRP1 in melanoma cells is barely detectable due to the action of SOX10/miR-338, this downregulation loses its effect in response to targeted therapy, allowing for NRP1 upregulation and drug resistance formation [200]. Accordingly, in carcinoma cells lacking the SOX10/miR-338 regulatory mechanism, miR-338 does not appear to be associated with drug resistance [200].

Similar to miR-338 in melanoma cells, in medulloblastoma cells, miR148a [249] and in non-small cell lung cancer, miR-152 inhibits the translation of NRP1 [268], while in cholangiocarcinoma, miR320 negatively regulates NRP1 expression [71]. Also by downregulating NRP1 levels, miR-376a inhibits in breast cancer cells the Wnt/β-catenin signaling axis and, thus, proliferation, migration, and invasion, and it promotes apoptosis [269].

In glioblastoma multiforme, miR-124-3p acts as a suppressor of NRP1, which promotes tumor cell proliferation and migration as well as tumor angiogenesis via PI3K/AKT/NFκB signaling [270]. In contrast, loss of miR-331-3p expression contributes, at least in part, to tumor growth and progression through upregulation of NRP2, and consequently increased proliferation and clonogenic growth [271]. In a rat glioma cell line (L9), miR-15b downregulated NRP2 expression and reduced angiogenic tube formation by attenuating MEK/ERK signaling [272]. Similarly, miR-486-5p acts as a tumor suppressor in colorectal carcinoma by downregulating NRP2 [273].

## 6. NRP in the Tumor Biological Setting

### 6.1. Tumor Cells and Tumor Microenvironment

NRP expression levels correlate with tumor growth, invasiveness, angiogenesis, and poor prognosis [95]. Cancer cells drive tumor progression and create a microenvironment which supports their growth. Various tumor cells express NRPs with diverse functions attributed to them. NRP1 promotes metastasis in melanoma [274]. It promotes dedifferentiation of cells and even causes CSCs to retain their stem cell properties [244,250]. Binding of VEGF-A to NRP1 favors growth and metastasis of solid tumors, whereas, binding of SEMA3A is generally associated with less migration and invasion of tumor cells and thus better prognosis [275,276]. The antagonism of SEMA3A and VEGF-A in leukemia cells is based on the fact that NRP1 preferentially binds SEMA3in a VEGF-competing manner [277].

Furthermore, Nrp1 promotes EMT via TGF-β, HH, and HGF/Met signaling [172], thereby increasing cell migration and invasion, which both contribute to metastasis [278]. The SEMA3F receptor NRP2 is upregulated by TGF-β1 in lung cancer and contributes significantly to TGF-β1-induced EMT [279]. NRP2B promotes TGF-β-triggered non-small cell lung cancer cell migration and invasion in vitro and metastasis in vivo [94]. In addition, NRP2B enhances HGF-induced AKT phosphorylation, and inhibition of MET reduced tumor cell migration [94]. These effects are independent of GIPC1 binding and PTEN recruitment [94].

Tumor cells have been in the scientific and therapeutic focus for long [280]. Doubtlessly, this is important. However, during the last two decades the scientific view on solid tumors has expanded, as the tumor mass not only consists of cancer cells, but also contains other cell types, such as resident stromal fibroblasts, resident and invading immune cells, and ingrowing ECs [281,282,283,284,285]. Under the influence of the neighboring tumor cells, these cells develop from bystanders to very active cells that even support tumor progression [281,286,287,288]. As an example, in the vicinity of tumor cells, fibroblasts undergo a differentiation process into cancer-associated fibroblasts (CAFs) by enlarging their actomyosin system and by exerting higher forces onto the stromal ECM [287,289,290]. Thus, they resemble myofibroblasts, which are typically found in wound tissue and help to regenerate the tissue [282,291]. The appearance of CAFs, in tumor tissue, similar to myofibroblasts in (chronic) wounds has been pinpointed in the phrase, that tumors are ‘wounds that never heal’ [292]. Soluble growth factors and immobilized ECM proteins, including their crosslinkage-related stiffening and cell-mediated tension, contribute to a mutual interplay between tumor cells and fibroblasts [281,282,293,294,295]. The soluble TGF-β, which is also tethered to and thus regulated by the ECM in a tension-dependent manner, is a key player in CAF differentiation. Consequently, CAFs produce additional growth factors, synthesize and deposit ECM proteins, and influence the metabolic milieu of the extracellular space within the tumor mass by taking up metabolic waste products and buffering protons [290,296,297]. All these steps promote survival and proliferation of cancer cells. Thus, tumor cells together with CAFs and other non-tumorigenic cells determine the tumor microenvironment, which in turn sustains the differentiation state of the latter. Moreover, the CAF-deposited ECM inter alia promotes tumor cell invasion and metastatic spreading [298]. NRP1 in CAFs promotes the assembly of fibronectin in an integrin-dependent manner. As part of the tumor-typical desmoplasia, increased fibronectin deposition results in ECM stiffness and NRP1-knockout in CAFs impairs tumor progression [141].

Dendritic cells and other antigen presenting cells, which surveil the tumor tissue, express NRPs [6,57]. Even if present in the tumor microenvironment, they do not trigger a major immune response. Immunosuppressive cytokines, TGF-β and IL-10, support the differentiation of the anti-inflammatory phenotype M2 of macrophages, which are typically found in the tumor microenvironment. M2-macrophages, also called tumor-associated macrophages (TAMs), express both NRPs. The expression of NRP2 in M2-macrophages correlates with their ability to carry out efferocytosis, a process in which apoptotic cells, e.g., tumor cells, are engulfed by macrophages without eliciting inflammation and a potential immune response against tumor cell components [299]. Thereby, M2 macrophages facilitate tumor progression in a NRP2-dependent manner. Other myeloid cells, such as myeloid suppressor cells, express NRPs and thus also contribute to the immunosuppressive conditions of the tumor microenvironment [6]. Moreover, M2 macrophages not only secrete anti-inflammatory cytokines, such as IL10, IL4 and TGF-β, thereby perpetuating differentiation of CAFs and M2 macrophages, but also promote the lymphoid cell population of regulatory T-cells, T_reg_s (CD4^+^CD25^+^Foxp3^+^ T-cells). In the tumor microenvironment, TAMs express SEMA4D on their surface, which forms a cell-cell contact with NRP1 on T_reg_s and activates them and other cells, such as ECs [6,300,301]. Moreover, by interaction with SEMA3A and plexinA4, NRP1 enhances immunoinhibitory signaling [48,92,184]. T_reg_s characteristically express NRPs, via which they crosstalk with the different cells of the innate and adaptive immunity. Thus, they orchestrate immunosuppressive processes [302]. Conspicuously, the vast majority of tumor-infiltrating T_reg_ cells express abundant NRP1, and the increased expression of NRP in T_reg_s correlates with poor prognosis, presumably because of its immunosuppressive properties against other CD4^+^-T_helper_ cells, CD8^+^ cytotoxic T-cells and natural killer cells [59,170]. Blunting this NRP-mediated immunosuppressive condition in the tumor microenvironment might be a strategy for immunotherapy in cancer, along with the recently developed immune check point inhibitors [303].

Another cell type within solid tumors are ingrowing ECs. Their diverse interactions with each other and with tumor cells involving NRPs are highlighted in the following sections. In tumors, NRP1 is involved in evading the control by the immune system and also in angiogenesis and formation of tumor-characteristic vasculogenic mimicry (VM) vessels that significantly contribute to resistance to antiangiogenic therapy [274].

### 6.2. Origin and Structure of the Tumor Vasculature

NRPs are involved in the (patho) physiological regulation of (lymph) angiogenesis [304]. Initially, cancer growth does not depend on blood supply, as the cells can be sufficiently supplied with oxygen and nutrients via diffusion [305]. As soon as a tumor grows beyond a few millimeters, it flips an angiogenic switch that triggers an angiogenic cascade in order to supply the tumor tissue with oxygen and nutrients and sustain its growth [306,307]. The vasculature becomes chronically activated, sustaining neovessel formation and thus exponential tumor growth [308]. Tumor angiogenesis is driven by numerous cytokines and chemokines, some of which are secreted by tumor-associated macrophages coming from bone marrow-derived monocytes, and by ECM-derived matrikines [309] and references therein]. Unlike healthy vasculature, the tumor vasculature is intrinsically leaky [310]. Also, in contrast to normal blood vessels, blood flow is disturbed in poorly differentiated, chaotically arranged, tortuous, and dilated tumor vessels [311]. The unexpectedly complex vascular system of tumors can be categorized into at least six types [310]: Feeder arteries and draining veins are enlarged and tortuous vessels coated with smooth muscle cells that connect the tumor with the vasculature of the body. So-called mother vessels are dilated and tortuous sinusoids with a thin and pericyte-poor wall, which develop from preexisting microvessels after detachment of pericytes and basement membrane degradation. They can convert to glomeruloid microvascular proliferations in which a tangle of finest vessels embedded by disordered pericytes and multilayered basement membrane structures. Other large vessels that are irregularly coated with smooth muscle cells are termed vascular malformations. While tumor capillaries resemble normal ones, sometimes blood-filled so-called vasculogenic mimicry channels can be found, that are lined not by ECs but by tumor cells instead [310].

**Figure 4:**
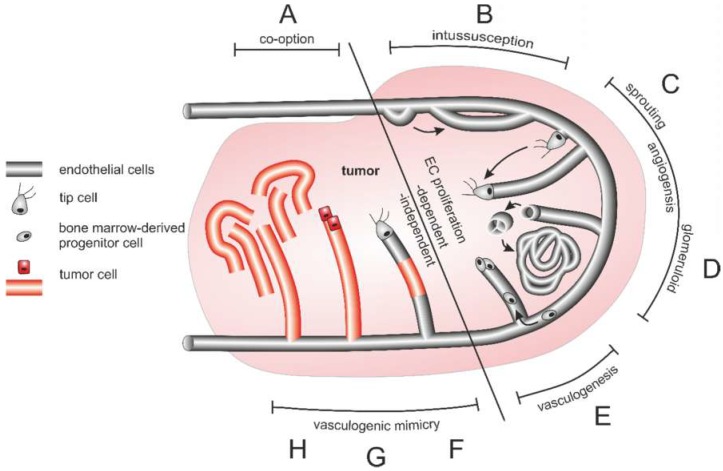
Different origins of the tumor vasculature. (**A**) Vessel co-option, (**B**) intussusception (**C**) sprouting angiogenesis, (**D**) glomeruloid angiogenesis, (**E**) vasculogenesis from bone marrow-derived progenitor cells, and vasculogenic mimicry in the form of (**F**) composites vessels, as well as (**G**) tubular type and (**H**) patterned type vasculogenic mimicry occur side by side and can also merge into each other. Of importance for tumor therapy is that vessel cooption and vasculogenic mimicry are independent of endothelial cell proliferation in contrast to intussusception, angiogenesis and vasculogenesis.

The multifaceted tumor vasculature can arise in various ways (Figure 4): Essentially, the tumor vasculature becomes permanently activated and forms new vessels from preexisting ones by sprouting of EC strands that form a lumen and anastomose. By recruitment of pericytes and smooth muscle cells, and formation of a new basement membrane these newly formed vessels are finally stabilized [308]. In addition to sprouting angiogenesis, tumor vascularization can take place by vessel co-option, intussusception, vasculogenesis, and vasculogenic mimicry [309]. Proliferation of tumor cells along existing vessels occurs at all tumor stages but predominantly early in tumor growth and is referred to as vessel cooption [312,313,314]. Neovascularization by intussusception is likewise more energy saving and faster than sprouting angiogenesis [315,316,317]. Here, the lumen of a preexisting vessel is split by EC columns growing into the lumen and expanding to form a new vessel wall. This occurs among others in gliosarcoma multiforme, melanoma, breast and colorectal cancer [315,316,317]. Tumor vessels can also be formed vasculogenically by recruitment of bone marrow-derived EPCs that differentiate into ECs [305,318,319,320]. Moreover, tumor endothelial cells (TECs) can be of different origin and thus very heterogeneous [321]. In vasculogenic mimicry, the blood supply of tumor tissue can furthermore be supported by conduits in which ECs are only partially or not at all involved. VM is significantly promoted by hypoxia and is associated with a transdifferentiation of CSCs, increased cell plasticity and facilitated metastasis [322,323,324]. As a result, VM channel-lining tumor cells phenotypically mimic ECs. These differ from normal ECs in their expression of NRP1, TIE-1, VEGF-C, endoglin, TFPI1, LAMC2, and EphA2. They also distinctly lack TIE-2, VEGFR1, VEGFR2, P-selectin, VCAM-1, and CD31 [322].

A variety of factors, especially the tumor microenvironment, determines the type of vascularization in the tumor mass [309,325]. Conversely, the morphology of the vasculature also strongly influences the tumor microenvironment [326].

### 6.3. NRP on Tumor Vessels

NRP1 is essential for VGFA-triggered and angiogenesis-inducing signaling in ECs and in tumor cells [13]. In particular, it is essential for tip cell morphology during sprouting angiogenesis [327]. To promote tip cell formation, the stalk cell phenotype has to be actively suppressed via NRP1, which limits Smad2/3 phosphoryation by activin receptor-like kinases ALK1 and ALK5 in response to TGF-β abd Bmp9/10, whereas Notch signaling downregulates NRP1 and promotes the stalk cell phenotype via activation of ALK1 and ALK5 [203]. Notch inhibition promotes expression of NRP1, which VEGFR2-independently regulates tip and stalk cell selection [203]. NRP1 suppresses stalk-cell-promoting SMAD2 and SMAD3 activation downstream of ALK1 and ALK5. Activation of ALK receptors cooperates with Notch to enhance HES and HEY expression, which regulate tissue-specific transcription factors and thus promote a tip cell phenotype [111,203,328]. Since tumor angiogenesis is dependent on tip cell formation, which is essentially modulated by the interaction of Notch and NRP1-mediated Smad2/3 signaling, inhibition of NRP1 is an attractive target for restraining tumor angiogenesis [203].

In developmental angiogenesis of mice, plexinD1 mediates signals from Sema3E, –A, and –F [63]. NRP/SEMA3 signaling significantly regulates tumor (lymph) angiogenesis and metastasis [329,330]. Lymphatic pericytes produce NRP1 that binds EC-derived SEMA3A. This Nrp1-SEMA3A interaction is essential for the stabilization of the vessel wall by pericytes [331]. Tumor cells can disrupt pericyte-EC interactions and thereby trigger their own metastasis [220]. SEMA3A is expressed in ECs, where it endogenously inhibits angiogenesis by signaling via NRP1 and plexinA1/A4 [133]. It is lost during tumor progression, and its reintroduction in a murine tumor model triggers apoptosis in ECs and subsequently in tumor cells, resulting in reduced vascular density and enhanced pericyte coverage of tumor blood vessels, i.e., in vessel normalization, and concomitant inhibition of tumor growth [133].

SEMA3C is released by pericytes and SMCs and counteracts VEGF-triggered angiogenic signaling in neighboring ECs and, thus, attacks immature vessel sprouts in pathological angiogenesis, rather than quiescent ECs in established vessels [209,332]. This selective pruning of immature vessels indicates different vascular-type specific SEMA3C holoreceptor compositions. SEMA3C exerts its inhibitory effect especially on ECs in immature vessel sprouts, as these markedly express NRP1 and PlexinD1; thus, specific plexin D1 ligands may be useful to inhibit tumor angiogenesis [332].

While SEMA3C inhibits tumor lymphangiogenesis and metastasis, its furin-cleaved form p65-SEMA3C has tumor-promoting properties in NRP2-expressing cancer cells, which was found at least in vitro, however, not yet in vivo [333,334,335,336,337]. Its direct binding to plexinB1 probably accounts, at least in part, for the pro-tumorigenic properties of p65-SEMA3C. In lymphatic ECs, NRP2 forms class-3 semaphorin holoreceptors with plexins A1 and D1 [138]. Furin-resistant SEMA3C inhibits in lymphatic ECs VEGF-C-triggered signaling and proliferation and, moreover, it promotes caspase-3-independent apoptosis in lymphatic ECs [333]. Additionally, M2 macrophage-assisted angiogenesis is inhibited by furin-resistant SEMA3C [333,338]. As cleavage of SEMA3C by another protease, ADAMTS-1 (a disintegrin and metalloproteinase with thrombospondin motifs), stimulates tumor cell migration, proteolytic cleavage seems to be a general principle for the modulation of semaphorin signaling [332,339].

An important aim in tumor treatment is a normalization of the tumor vasculature, rather than inhibition of angiogenesis, so that hypoxia is reduced by improved perfusion and drugs can reach tumor cells better [311,340]. SEMA3 controls cellular functions in various cell types within the tumor vasculature and the tumor microenvironment. This fact can be used to increase the effectiveness of other cancer therapies by vascular normalization [63]. By binding to NRP1, secreted SEMA3 induces the formation of holoreceptor/ligand complexes with plexin, signaling of which promotes vessel normalization [63,133,225,341]. On the other hand, binding of SEMA3 to NRP1 also has adverse side effects, such as attraction of tumor-promoting macrophages and increase in vascular permeability [342,343]. A parenterally administrable SEMA3A point mutant (A106K_ΔIg-b) that does not bind to NRP1 strongly binds to plexin A4, unlike its wild type form [344]. In pancreatic carcinoma and RIP-Tag2 mouse models, it reduces vascularization, inhibits tumor growth, and metastasis, and also improves accessibility and effect of conventional chemotherapy with gemcitabine (2’,2’-difluoro 2’deoxycytidine), while in a mouse model of age-related macular degeneration, it inhibits retinal neovascularization [344].

### 6.4. NRP-Dependent Effects of Tumor Cells on Endothelial Cells

In tumors, NRP enhances angiogenesis probably by blocking receptor endoytosis via trans-cellular interactions and thereby stabilizing receptor signaling [168]. In this way, NRP1 on non-ECs can inhibit angiogenesis and reduce the initiation of tumor growth by altering VEGFR2 internalization and signaling [168]. In dormant tumors of apparently healthy individuals carrying microscopic tumors and dysplastic foci, which do not develop without angiogenesis for many years, this may be an essential mechanism [345]. In pancreatic duct adenocarcinoma as well as in murine fibrosarcoma, formation of such NRP1/VEGFR2 trans-complex reduces vessel branching and proliferation of tumor cells [68].

Independent of VEGFR1, PlGF promotes invasion and VM in melanoma via NRP1 [274]. Unlike ECs, VM-lining tumor cells express NRP1, VEGF-C, TIE-1, endoglin, tissue factor pathway inhibitor (TFPI1), laminin subunit γ2 (LAMC2), and EphA2, whereas they do not express VEGF receptors -1 and -2, TIE-2, CD31, vascular adhesion protein-1 (VCAM-1) and P-selectin [322]. VM as well as tumor cell invasiveness correlates with increased NRP1 expression due to upregulation of VEGF-A, secretion of matrix metalloproteinase (MMP)-2 and -9, and activation of αvβ5 integrin [77,346].

## 7. NRP as a Therapeutic Target

In cancer cells, signaling cascades are often activated by the constitutive activation of an oncogene, upon which they become dependent (‘oncogene addiction’), and on which a therapy inhibiting this signaling can be based [347]. Unfortunately, such targeted therapies typically lose their efficacy through adaptive mechanisms in the cancer cells, e.g., by upregulation of parallel signal cascades that promote tumor cell survival and proliferation [348]. Thus, in place of the pathway on which the tumor is dependent, tumor cells increase expression and activity of RTKs, such as EGFR, MET, and FGFR, by cytokines and growth factors in the tumor microenvironment to escape tumor therapy [349].

Since NRPs can associate with diverse receptors into holoreceptors, they are promising targets for tumor therapies, and development of highly specific and highly potent NRP1 inhibitors is of outstanding interest [64,350]. In addition, NRP1 has been suggested as a biomarker candidate to assess the use and success of VEGF/VEGFR targeting agents [351]. However, no significant correlation was found between NRP1 expression and response to treatment with the VEGF antibody bevacizumab or survival of patients with astrocytoma or ovarian cancer [352,353]. On the other hand, according to the BATON-CRC study, low NRP1 levels are associated with better progression-free survival in patients treated with the tyrosine kinase inhibitor, tivozanib [354]. 

NRP1 is generally considered as a tumor-promoting coreceptor, but the situation is not entirely clear [355]. In colon cancer, elevated NRP1 expression is associated with a less severe prognosis [356], and, at least in PANC-1 pancreas adenocarcinoma cells, NRP acts as a tumor suppressor [355]. Following the prevailing view that NRP1 is a tumor promoter, several studies have been performed to test NRP1 as a therapeutic target [127]. In addition, NRP1-coated magnetic nanoparticles may be useful in diagnosis and therapy of gliomas [357].

### 7.1. Soluble NRP in Tumor Therapy

The use of soluble sNRP as a decoy receptor has been investigated in animal models in tumor therapy. sNRP1 caused extensive hemorrhage, damaged vessels, and apoptotic tumor cells in tumors of rat prostate carcinoma cells and inhibited tumor angiogenesis and growth in murine granulocytic sarcoma (chloroma) [23,358]. Likewise, in a systemic leukemia mouse model, dimerized sNRP1 led to a decrease in circulating leukemia cells and reduced infiltration of the liver and spleen, as well as lessened neovascularization and cellularity in the bone marrow, resulting in significantly increased survival time [358].

### 7.2. NRP-Directed Antibodies

Blocking NRP1 with antibodies selectively inhibits angiogenesis and in combination with anti-VEGF therapy, it further reduces tumor growth, suggesting that NRP1 antibodies may render tumor vessels more responsive to anti-VEGF therapy [107].

NRP1 is overexpressed by angiogenic ECs of the tumor vasculature and in diverse tumor cells [359,360,361]. However, since NRP1 is expressed in addition to tumor tissue in various tissues, a NRP1-directed antibody is rapidly eliminated, interfering with physiological tissue functions and also reducing its uptake by tumor cells [362].

A monoclonal antibody against NRP1 inhibits EC migration and tumorigenesis in mouse tumor models [107,131]. Targeting the b1/b2 tandem domain of NRP1 with a monoclonal antibody results in a reduced density of the vascular network lacking associated pericytes, and delays tumor growth only in combination with anti-VEGF therapy, but is ineffective when used alone [107].

In renal cancer, angiogenesis inhibition by blocking NRP1/VEGFR2-mediated signaling with bevacizumab in combination with the RTK inhibitor sunitinib had toxic hematologic and vascular side effects and caused hypertension [363].

A phase I trial with a human monoclonal IgG1 antibody (MNRP1685A) targeting NRP1’s VEGF-binding domain was promising, but another phase Ib trial with concomitant inhibition of NRP1 and VEGF in combination with chemotherapy showed an unexpectedly high proteinuria and toxicity [364,365]. Thus, concomitant blocking of NRP1 and VEGF may be limited by toxicity [366].

### 7.3. Targeting NRP with Peptides/Small Molecule Inhibitors

The first NRP-specific small molecule peptide inhibitor is EG00229 (HY-10799) [367]. Similarly, the peptide A7R (ATWLPPR) specifically binds to NRP1, has anti-angiogenic activity in vitro by inhibiting NRP1/VEGFR2 signaling, and curbs tumor angiogenesis and tumor growth in vivo [174,368]. The synthetic NRP1-targeting peptide EG3287 blocks VEGF signaling and induces apoptosis of NRP1-expressing tumor cells expressing [369]. Also cyclic peptides, such as vasotide (a retro-inverted peptidomimetic, _D_CLPRC), DG1 (CRRPRMLTC) and DG2 (CRSRRIRLC) have suppressive effects on angiogenesis, tumorigenesis, and invasion, respectively, in preclinical mouse and primate models of human retinal diseases [370] and in a xenograft mouse model of non-small cell lung cancer [370,371]. The 28 C-terminal amino acids of VEGF-A, N-terminally derivatized with octanoic acid, termed EG00086, also efficiently binds to and inhibits NRP1 [372].

Distinguishing between the two NRP isoforms, the small molecule inhibitor EG01377 selectively binds to the arginine-binding pocket of NRP1 without binding to NRP2, and it functionally inhibits VEGF-A-induced angiogenesis, cell migration, melanoma cell invasiveness, and T_reg_ cell activation with good stability in vivo [373].

Based on the inhibitory peptide KPPR, branched pentapeptides in which the K side chain has been extended by an additional homoarginine have been developed, which are up to 30 times more active than ATWLPPR with respect to their inhibition of the interaction of VEGF-A_165_ with NRP1 [367,368,374]. By additionally replacing the first P by L-2,3-diaminopropionic acid or L-2,4-diaminobutyric acid residue, the half-life in the plasma increases to a considerable 34 or 41 h [374].

Targeting the transmembrane domain of NRP1, a peptide corresponding to the NRP1 transmembrane domain, termed pTM-NRP1, shows antiangiogenic activity in a xenograft mouse model of glioma and inhibits breast cancer growth and metastasis [375,376].

The NRP1-binding motif has been harnessed to generated peptides that are directed to and penetrate into the tumor. Such NRP1-specific tumor targeting peptides effectively enhance the uptake of various therapeutic agents [377]. These peptides follow the C-end rule (CendR) with the consensus sequence R/KXXR/K, which shows optimum binding affinity when two non-basic amino acids are between the flanking R/K [40,378]. Systemically administered tumor-penetrating peptides home to and penetrate the tumor tissue in several steps [379]. First, they are recruited to tumor ECs and tumor cells by their interaction with integrins, such as αvβ3 and αvβ5. After subsequent proteolytical unmasking of the CendR motif by the tumor-related urokinase plasminogen activator (uPA) and binding to NRP1, the peptides are micropinocytotically taken up and, subsequently, by a transcytosis cascade, distributed deep into the tumor parenchyma [242]. NRP 1 colocalizes with SNX5, which is a marker of macropinocytosis, but not CLCa, which is a marker for clathrin-mediated endocytosis [380]. Enhancing survival and reducing metastasis, in an orthotopic mouse model of pancreatic ductal adenocarcinoma, irinotecan-loaded and gold-labeled silica nanoparticles co-administered with iRGD are transcytotically conveyed in vesicles from the blood vessel lumen to perinuclear regions within cancer cells as demonstrated by electron microscopy [381]. Triggering the CendR pathway with tumor-penetrating peptides allows the penetration of coupled or co-administered drugs or nanoparticles into tumors [382]. Interestingly, drugs need not be covalently bound to tumor-penetrating peptides for uptake by TECs and tumor cells [243].

Dual targeting compounds, such as the peptide RGD-ATWLPPR, encompassing ligands of NRP1 and integrin αvβ3 that are both overexpressed in the tumor microenvironment accumulate with higher specificity in the tumor and, unlike uncoupled NRP1 and integrin ligands, reduce turnover and/or surface internalization of NRP1 [383].

A phase 1 clinical trial of the integrin-binding, tumor tissue-homing peptide CEND-1 (i.e., iRGD; CRGDKGPDC) in combination with nanoparticle albumin bound (nab)-paclitaxel and gemcitabine in metastatic exocrine pancreatic cancer has started this year [384]

LD22-4 is an N-terminal fragment of FGF2 that binds with its CendR motif-corresponding C-terminus (KDPKR) to NRP1 and inhibits in vitro migration of ECs, tumor cells, and fibroblasts, and in vivo, it suppresses tumor angiogenesis and growth in animal models of breast, prostate, and lung carcinoma without being cytotoxic, inducing apoptosis, or affecting cell proliferation rates [385,386,387].

An immunoglobulin FC-fused tumor tissue penetrating peptide, Fc-TPP11 (HTPGNSKPTRTPRR), binds to the VEGF-binding site of NRP1 with 1000-fold higher affinity as compared to NRP2 [388]. After binding to NRP1 it is internalized and enhances vascular and paracellular permeability in tumors by downregulating VE-cadherin [388]. In vivo, it suppresses VEGF-dependent angiogenesis and inhibits tumor growth [388]. FC-TPP11 also promotes the efficacy of co-administered doxorubicin, and its coupling to cetuximab (Erbitux), a monoclonal antibody against EGFR, significantly improves its tumor penetration and accumulation without compromising its serum half-life [388].

The disulfide-bridged TT1 (CKRGARSTC) and its linear analogon LinTT1 (AKRGARSTA) home to breast cancer in mouse models and enhance the antitumor potency of coadministered therapeutics [389,390]. After binding to p32 (GClqR), which is expressed on peritoneal caricnoma cells of gastric, ovarian and colon origin in mouse models as well as clinical peritoneal carcinoma explants, cleavage by uPA exposes their NRP1 targeting CendR motif which mediates vascular exit and tumor penetration [391].

Docetaxel-loaded nanoparticles that dually target NRP1 and CD44 with the tLyP-1 peptide (CGNKRTR) and hyaluronic acid, respectively, home in on metastatic tumor cells and metastasis-supporting neovasculature and suppress tumor cell invasion and inhibit lung metastasis in three mouse models of triple-negative breast cancer [392].

Diagnostically, lipid microbubbles with conjugated NRP1-targeted peptides CRPPR and ATWLPPR specifically bind to NRP1-expressing cells and allow ultrasound imaging of angiogenic tumors [361].

Theranostically, polysiloxane nanoparticles combining a magnetic resonance imaging (MRI) contrast agent, a photosensitizer and the NRP1-targeting motif KDKPPR can be employed to destroy tumor neovessels in glioblastoma, because they accumulate in the tumor vessel wall and show no cytotoxicity unless after exposure to light during photodynamic therapy [393].

CendR peptides also bind to NRP2 and are subsequently internalized [394]. A truncated and thus noncyclic tumor-homing peptide tPlP-1 shows a promising possibility for targeting therapeutic and diagnostic agents to breast cancer cells, as it binds to both NRP1 and NRP2 and improves extravasation of co-injected nanoparticles into the tumor tissue [394].

The risk that tumor-penetrating peptides might also promote the spread of metastases by inducing increased vascular permeability at the tumor site, in addition to improved drug permeation, appears to be limited, as they not only not increased metastasis but also reduced them [377,395,396]. Moreover, there is no evidence that systemically administered tumor-penetrating peptides affect the vasculature of normal tissues [377].

### 7.4. Natural Compounds That Target NRP

In addition to reducing intratumoral hypoxia and elevated oncotic pressure and improving the accessibility with chemotherapeutic agents by normalization of the tumor vasculature, as a further possible approach, a tumor-specific vessel disruption in combination with chemotherapeutic agents is also conceivable for some cancers. In xenograft fibrosarcoma and epidermoid carcinoma mouse models, intravenously injected NRP1-specific rhodocetin-αβ selectively destroys the vasculature only in tumors and results in hemorrhage, but not in other tissues [167].

**Figure 5:**
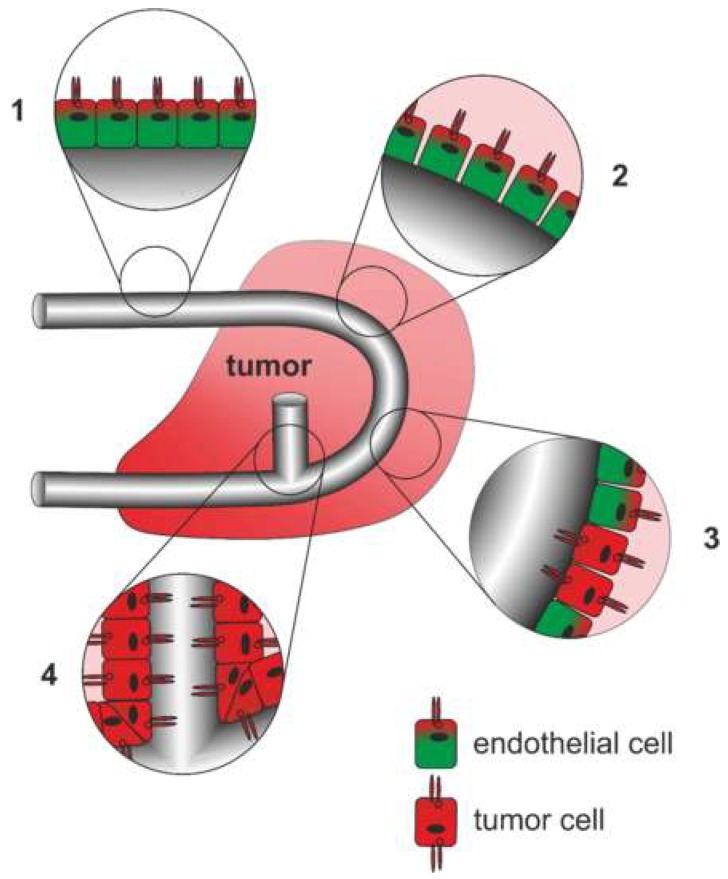
Accessibility of neuropilin-1 in different types of tumor vessels. (**1**) In normal endothelium, NRP1 (highlighted in red), located on the basolateral side of ECs (green), is inaccessible from the bloodstream. (**2**) In tumors, the endothelium is generally leaky. Therefore, when viewed from the bloodstream, NRP1 is no longer inaccessible on the basolateral side. (**3**) In composite vessels of the tumor vasculature, patches of ECs are replaced by tumor cells that have acquired an EC-like phenotype. They have NRP1 on their entire surface, making NRP1 easily accessible. (**4**) When vasculogenic mimicry occurs, tubes formed by tumor cells are directly connected to the bloodstream. At such sites, NRP1 is particularly accessible from the bloodstream.

As in normal arteries, NRP1 occurs on the basolateral side of TEC of intrinsically leaky tumor vessels [167]. Moreover, it is exposed to the bloodstream in composite vessels, where tumor cells that have adopted an EC phenotype replace ECs in varying degrees. In some cancers vasculogenic mimicry has been observed, where vessel-like tubes that are completely lined by tumor cells contribute to the blood supply of the tumor. Such composite and VM vessels present NRP1 to the blood stream and are susceptible to NRP1 targeting compounds (Figure 5) [167].

Binding to the b1/b2 domain of NRP1, rhodocetin-αβ triggers NRP1/MET signaling only in cells that expose NRP1 to the bloodstream [166,167]. Therefore, development of novel NRP1-targeting lead structures starting from the C-type lectin-related rhodocetin-αβ may be worthwhile.

Lebein, a snake venom disintegrin from *Macrovipera lebetina* inhibits tumor angiogenesis by reducing the expression of NRP1 and VEGF in a quail embryonic chorio-allantoic membrane system as well as in a human colon adenocarcinoma xenograft mouse model [397].

## 8. Conclusions

NRPs, as coreceptors of important RTKs, integrins, and other receptors, are of paramount importance for formation and functioning of the tumor vasculature. In this context, NRPs modulate cellular responses by capturing ligands, regulating growth factor expression, endocytosis and recycling, and by signaling independently. The complex interplay of different cell types within the tumor microenvironment causes dysregulated angiogenic signaling resulting in pathological tumor angiogenesis. The highly irregular shape and comparatively poor functionality of the tumor vasculature complicates treatment with drugs administered via the bloodstream. To promote tumor therapy with cytostatic drugs, vessel normalization is sought. NRPs represent a potential therapeutic target due to their multifaceted roles and the fact that they are highly expressed on tumor ECs and tumor cells. As NRP also plays a key role in the uptake of nutrients by cells, NRP appears to be particularly suitable for introducing drugs into both TECs and tumor cells.

## Abbreviations

3′-UTR	3′ untranslated region
ADAM	A disintegrin and metalloproteinase
AGO	Argonaute
AKT	Protein kinase B
ALK	Activin receptor-like kinase
BMP	Bone Morphogenetic Protein 1
BRAF	Rat/rapidly accelerated fibrosarcoma, isoform B
CAF	cancer-associated fibroblasts
CD	Cluster of differentiation
CendR	Carboxy-terminal end rule
CSC	Cancer stem cell
CUB domain	Cubilin homology domain
Dlg domain	Discs-large domain
EC	Endothelial cell
ECM	Extracellular matrix
EGF(R)	Epidermal growth factor (receptor)
EMT	Epithelial to mesenchymal transition
ErbB	Erythroblasotsis oncogene B
ERK	Extracellular-signal-regulated kinase
FGF(R)	Fibroblast growth factor (receptor)
EphA2	Erythropoietin-producing human hepatocellular (EPH) receptor A2
FAK	Focal adhesion kinase
Frzb	Frizzled-related protein
GAIP	G alpha interacting protein
GAP	GTPase activation protein
GIPC	GAIP interacting protein, C terminus
GIPC1	GIPC PDZ domain containing family member 1, synectin
GLUT1CBP	Glucose transporter 1 C-terminal binding protein
Gq	Guanine nucleotide-binding protein, q polypeptide
GLI1	Glioma-associated oncogene homolog 1
Her2	Human epidermal growth factor receptor 2
HGF(R)	Hepatocyte growth factor (receptor)
HH	Hedgehog
IIP1	insulin-like growth factor-1 receptor-interacting protein 1
Jnk	c-Jun N-terminal kinase
L1CAM	L1 cell adhesion molecule
LAMC2	Laminin subunit γ2
LRP5	Low-density lipoprotein receptor related protein 5
MAM domain	meprin/A5-protein/PTPmu
MAP(K)	Mitogen-activated protein (kinase)
MET	Mesenchymal-epithelial transition factor (MET) proto-oncogene, Hepatocyte growth factor receptor, HGFR
miR	microRNA
MMP	Matrix metalloproteinase
NIP	Neuropilin-1 interacting protein
NRP	Neuropilin
p130Cas	CRK associated substrate
PDGF(R)	Platelet-derived growth factor(receptor)
PD-L1	Programmed cell death 1 ligand 1, CD274
PDZ bd	Post synaptic density/Disks large/Zonula occludens-1 binding domain
PlGF(R)	Placenta growth factor (receptor)
PI3K	Phosphoinositide 3-kinase
PKC	Protein kinase C
PSD-95 domain	postsynaptic density protein 95 domain
PTEN	Phosphatase and tensin homolog
PTPmu	receptor-type protein tyrosine phosphatase µ
RAS	Rat sarcoma
RhoGEF	Rho guanine nucleotide exchange factor 1
RTK	Receptor tyrosine kinase
sNRP	Soluble neuropilin
SAPK1	Stress-activated protein kinase 1
SEMA	Semaphorin
SEMCAP1	Semaphorin 4C (SEMA4C)-interacting protein 1
Src	Sarcoma
Syx	Synectin-binding GEF
TAM	Tumor-associated macrophage
TEC	Tumor endothelial cell
TFPI1	Tissue factor pathway inhibitor
TGF-β(R)	Transforming growth factor-β (receptor)
TIE	Tyrosine kinase with immunoglobulin-like and EGF-like domains
TIP2	Tax-interacting protein 2
TORC2	rapamycin-sensitive TOR complex 2
T_reg_	Regulatory T Cell
uPA	urokinase plasminogen activator
VCAM-1	Vascular adhesion protein-1
VEGF(R)	Vascular endothelial growth factor (receptor)
VM	Vasculogenic mimicry
WIF1	Wnt inhibitory factor 1
Wnt	Wingless-related integration site
YAP1	Yes-associated protein 1
ZO-1 domain	Zonula occludens-1 domain
